# Inverse iron oxide/metal catalysts from galvanic replacement

**DOI:** 10.1038/s41467-020-16830-4

**Published:** 2020-06-29

**Authors:** Yifeng Zhu, Xin Zhang, Katherine Koh, Libor Kovarik, John L. Fulton, Kevin M. Rosso, Oliver Y. Gutiérrez

**Affiliations:** 1 Institute for Integrated Catalysis, and Fundamental and Computational Science Directorate, Pacific Northwest National Laboratory, Richland, WA USA; 2 Physical and Computational Sciences Directorate, Pacific Northwest National Laboratory, Richland, WA USA; 3 Environmental Molecular Sciences Laboratory, Pacific Northwest National Laboratory, Richland, WA USA

**Keywords:** Catalyst synthesis, Heterogeneous catalysis, Materials for energy and catalysis, Synthesis and processing

## Abstract

Key chemical transformations require metal and redox sites in proximity at interfaces; however, in traditional oxide-supported materials, this requirement is met only at the perimeters of metal nanoparticles. We report that galvanic replacement can produce inverse FeO_x_/metal nanostructures in which the concentration of oxide species adjoining metal domains is maximal. The synthesis involves reductive deposition of rhodium or platinum and oxidation of Fe^2+^ from magnetite (Fe_3_O_4_). We discovered a parallel dissolution and adsorption of Fe^2+^ onto the metal, yielding inverse FeO_x_-coated metal nanoparticles. This nanostructure exhibits the intrinsic activity in selective CO_2_ reduction that simple metal nanoparticles have only at interfaces with the support. By enabling a simple way to control the surface functionality of metal particles, our approach is not only scalable but also enables a versatile palette for catalyst design.

## Abstract (web summary)

While typical catalysts involve oxide-supported metals, inverse catalysts of oxides on metal supports offer an attractive alternative. Here, authors prepare FeO_x_-coated Rh nanoparticles via galvanic replacement and dissolution-precipitation to form effective CO_2_ reduction catalysts.

## Introduction

Metal particles play a key role in chemical transformations that require activation of H_2_ or hydrogenation/dehydrogenation of substrates. In many cases, the metal particles provide only one step in the catalytic cycle. For instance, metals have low activity in CO_2_ reduction because of weak CO_2_ adsorption, whereas the polar surface of oxides readily adsorbs CO_2_ but suffers from low activity for H_2_ activation^1–3^. Thus, metal–oxide interfaces are much more effective because both the redox sites required to activate CO_2_ and the metals providing active H_2_ are in proximity. Challenges for maximizing such interfaces are stabilizing small metal particles on oxide supports^4–7^ or forcing migration of oxides onto metal particles while avoiding harsh synthesis conditions^8–12^.

Inverse catalysts—oxides supported on metals—offer an attractive alternative to overcome the constraints of typical supported metal catalysts because reactants can bind to sites in the oxide overlayer, onto the metal domains, or at their interface. Typically, surface science research selects only well-defined inverse catalysts to provide a basic understanding of their adsorption and catalytic properties; however, advancing from this approach into the more complex conditions relevant to technical applications is essential^13–16^. In this regard, a major obstacle is encountered because typical surface science approaches for preparing inverse catalysts, such as reduction at high temperature^12^, deposition in ultrahigh vacuum^1,13^, and deposition at atomic layers^17^, are challenging to scale beyond certain models.

We report here a simple galvanic replacement approach for generating inverse FeO_x_/metal nanostructures. During galvanic replacement, one metal dissolves as a sacrificial template while a different metal ion in solution is reductively deposited onto the template. This process is driven by the differences of reduction potentials of the redox pairs, allowing a single, simple, and low-temperature step for synthesis of nanostructures^18–23^. Following this, research has focused on preparing metals^18^, metal alloys^19,24^, oxides^21^, and metal–oxides^25,26^ with controllable shapes. In our case, the solid support undergoing oxidation—hyperstoichiometric and sometimes referred to as cation-excess or partially reduced magnetite (Fe_3_O_3.7_)^27^—supplies electron equivalents in the form of Fe^2+^ enriched at the oxide surface, which reduce Rh^3+^ or Pt^4+^, thereby depositing metal nanostructures (Eqs. (1)–(3)).

1 $${\rm{Rh}}^{3+} + 3e^ - \to {\rm{Rh}}.$$

2 $${\rm{Fe}}^{2 + } \to {\rm{Fe}}^{3 + } + {\rm{e}}^ -.$$

3 $${\rm{Rh}}^{3 + } + 3{\rm{Fe}}^{2 + } \to {\rm{Rh}} + 3{\rm{Fe}}^{3 + }.$$

We discovered that in addition to acting as sacrificial species, Fe^2+^ dissolves, and adsorbs onto the as-formed metal particles as Fe(II)-oxyhydroxide. The surface property of the metal is thus greatly changed by the FeO_*x*_ overlayer, endowing the nanostructure with the high density of active sites for CO_2_ reduction that well-dispersed Rh particles have only at the interface with Fe_3_O_4_. This yields activity and selectivity for CO production significantly higher than well-dispersed Rh particles without FeO_*x*_ overlayers. Our method demonstrates that the surface of metal nanoparticles can be manipulated by the sacrificial species during galvanic replacement, whereas galvanic replacement was previously thought to control only nanostructure morphologies.

## Results and discussion

### Identification of FeO_*x*_ overlayer on Rh

**Fig. 1:**
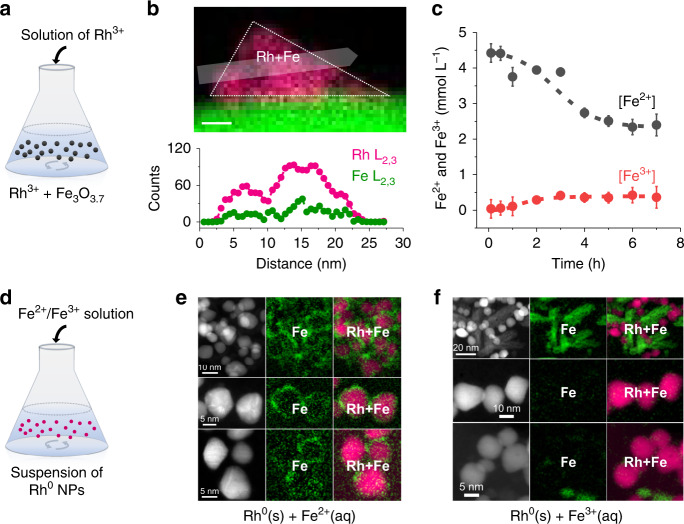
Synthesis of FeO_*x*_/Rh/Fe_3_O_4_ by galvanic replacement and reference experiments to elucidate the mechanism of FeO_*x*_ coating on Rh. Synthesis of FeO_*x*_/Rh/Fe_3_O_4_-fresh by galvanic replacement, where powder Fe_3_O_3.7_ is contacted with a solution containing Rh^3+^ (**a**). Rh and Fe L-edges EELS images of the FeO_x_/Rh/Fe_3_O_4_-fresh and the corresponding line-scan profile showing the FeO_x_ coating on Rh; the scale bar is 5 nm (**b**); more images can be found in Supplementary Figs. 2–5. Evolution of concentrations of aqueous Fe^2+^ and Fe^3+^ when Fe_3_O_3.7_ is contacted with the Rh^3+^ solution (**c**). Scheme of the reference experiments, where pre-formed Rh nanoparticles were contacted with solutions containing Fe^2+^ or Fe^3+^ (**d**). HAADF-STEM-EELS images of the solids produced after contacting Rh nanoparticles with Fe^2+^ (**e**) or Fe^3+^ (**f**) in solution showing the selective adsorption of Fe^2+^ on Rh producing the Fe(II)-oxyhydroxide adlayers on Rh.

We performed the synthesis by simply suspending Fe_3_O_3.7_ (“Methods” section and Supplementary Fig. 1 for synthesis) in aqueous RhCl_3_ solution (Fig. 1a), yielding the as-prepared material (FeO_*x*_/Rh/Fe_3_O_4_-fresh). High-angle annular dark-field scanning tunneling electron microscopy (HAADF-STEM) imaging of FeO_*x*_/Rh/Fe_3_O_4_-fresh (Fig. 1b and Supplementary Figs. 2–5) showed that the deposited nanostructures distribute along the whole surface of Fe_3_O_4_ with the average size of 6.6 nm. These nanostructures seem to be composed by smaller Rh nanoparticles of around 2 nm. The nanosized structures were further examined by electron energy-loss spectroscopy (EELS) while manipulating the sample to avoid overlapping with the support along *z*-axis. Maps of Rh L_2,3_ and Fe L_2,3_ edges (Fig. 1b and Supplementary Figs. 3–5) show Fe signals in regions of the Rh domains. The line profile indicates that significant amounts of Fe coincide with Rh particles. The Fe spectra from the Rh domains give a lower loss energy (by 0.7 eV) than the signal from Fe_3_O_4_ (Supplementary Fig. 3). This indicates that the Fe on Rh nanostructures have a lower average oxidation state (i.e., +2) than that in Fe_3_O_4_ (+8/3).

Rh K-edge X-ray absorption near edge structure (XANES, Supplementary Fig. 3) showed that the white-line of FeO_*x*_/Rh/Fe_3_O_4_-fresh is similar to that of Rh foil. Linear combination fitting indicated that 77 mol.% of Rh is metallic (Supplementary Fig. 3 and Supplementary Table 1). This agrees well with the Rh extended X-ray absorption fine-structure (EXAFS) fitting showing that the Rh species have high Rh–Rh coordination (Supplementary Fig. 6 and Supplementary Table 2). The fitting of the spectra required a Rh–O path with a coordination number of 1.9 ± 0.5. Thus, 23 mol.% of Rh remains oxidized, probably because of its interaction with the FeO_*x*_ species (Supplementary Table 1). The results indicated that inverse FeO_*x*_/Rh nanostructures were formed where Rh was reductively deposited while Fe(II)-oxyhydroxide species bind onto the Rh.

### Mechanism for the formation of FeO_*x*_ overlayer

We used the ferrozine method^28^ to monitor changes of Fe^3+^ and Fe^2+^ concentrations during synthesis of FeO_*x*_/Rh/Fe_3_O_4_-fresh (Fig. 1c). Fe^2+^ was released immediately after Fe_3_O_3.7_ was dispersed in the solution of Rh^3+^, which is consistent with the acidic Fe_3_O_4_ oxidation chemistry (Eq. (4))^27^ because Fe^2+^ is much more soluble than Fe^3+^, and the solution is initially free of aqueous Fe^2+^ upon first contact the Rh^3+^ solution. The pH was observed to initially drift upwards from ~4 to 5, which is consistent with consumption of protons during the release (see section of methods for pH changes).

4 $$	\left[ {\rm{{Fe}}^{3 + }} \right]_2\left[ {\rm{{Fe}}^{2 + }} \right]{\rm{O}}_{3.7}\left( {\rm{s}} \right) + 2{\rm{H}}^ + \left( {\rm{{aq}}} \right) \to \frac{3}{4}\left\{ {\left[ {{\rm{Fe}}^{3 + }} \right]_{\frac{8}{3}}\left[ {\rm{{Vacancy}}} \right]} \right\}{\rm{O}}_{3.7}\left( {\rm{s}} \right) \\ 	+ {\rm{H}}_2{\rm{O}} + {\rm{Fe}}^{2 + }\left( {{\rm{aq}}} \right).$$

The particle surface will be enriched in Fe^2+^ during the Fe^2+^ release into solution^29^, thereby maintaining a dynamic equilibrium^27^. In parallel, Rh^3+^ was reduced and deposited as the nanostructures that adsorb and bind Fe(II)-oxyhydroxide during the progressive Fe^2+^ accumulation on the Fe_3_O_4_ surface (see below). This leads to a gradual reversal of the reaction in Eq. (4), detectable by a pH decrease from ~5 to 2.5 and an increase in Fe^3+^ in solution, reaching equilibrium after 3 h synthesis time. Note that if Rh^3+^ and Fe^2+^ (Rh^3+^:Fe^2+^ = 1:3) were mixed at the conditions of the galvanic replacement, neither Rh nor FeO_*x*_ particles are observed by HAADF-STEM. Thus, Rh nucleation and growth requires the Fe_3_O_4_ surface and productions of Fe^2+^ and Fe^3+^ in solution follow different mechanisms.

Because formation of metallic Rh is accompanied by increasing detectable aqueous Fe^3+^ (Eq. (3)) and consumption of Fe^2+^ (Fig. 1c), we attribute the Fe(II)-oxyhydroxide coating on Rh particles to the dynamic equilibrium of the Fe^2+^ release process (i.e., the reverse of Eq. (4))^27,29^. To confirm the selective interaction of Fe^2+^ with Rh, we contacted pre-formed Rh nanoparticles with solutions containing either Fe^2+^ or Fe^3+^ cations and analyzed the recovered particles (Fig. 1d). The syntheses of these reference materials are described in the section of methods. The EELS images showed that Fe^2+^ species adsorb on the Rh surface to form a core-shell-like nanostructure (Fig. 1e), whereas Fe^3+^ species precipitate as a segregated phase with only weak association with Rh (Fig. 1f).

**Fig. 2:**
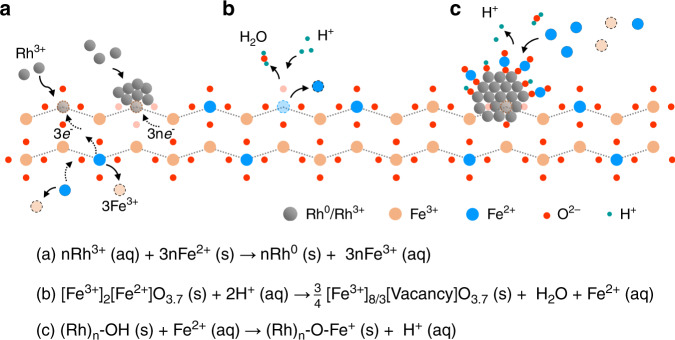
Mechanism for synthesis of inverse FeO_*x*_/Rh/Fe_3_O_4_. Formation of Rh nanoparticles on Fe_3_O_4_ and release of Fe^3+^ by galvanic replacement (**a**), dissolution of Fe^2+^ species (**b**), and selective deposition of Fe^2+^ on Rh particles (**c**).

Overall, the charge transfer of galvanic replacement consumes Fe^2+^ supplied by Fe_3_O_4_ for Rh^3+^ reduction yielding Rh particles (Fig. 2a). In parallel, Fe^2+^ released from the solid (Fig. 2b) adsorbs selectively on Rh (Fig. 2c). In order to verify the generality of our methodology to prepare inverse nanostructures, we also performed the galvanic replacement between the Pt^4+^ cations and Fe_3_O_3.7_. The HAADF-STEM-EELS showed that FeO_*x*_ species coat the Pt nanoparticles (Supplementary Fig. 7). Hence, during the synthesis of FeO_*x*_/metal nanostructures, the Fe^2+^ is not only a sacrificial species as one expects from the galvanic replacement alone, but a key constituent for tuning the surface of the metal nanoparticles. The method offers many possibilities to tune the properties and structures of the final materials by controlling the rates of the individual processes taking place during the synthesis. Further work to control the metal particle size and FeO_*x*_ coverage is ongoing.

### Comparison of FeO_*x*_-covered and bare Rh particles supported on Fe_3_O_4_

**Fig. 3:**
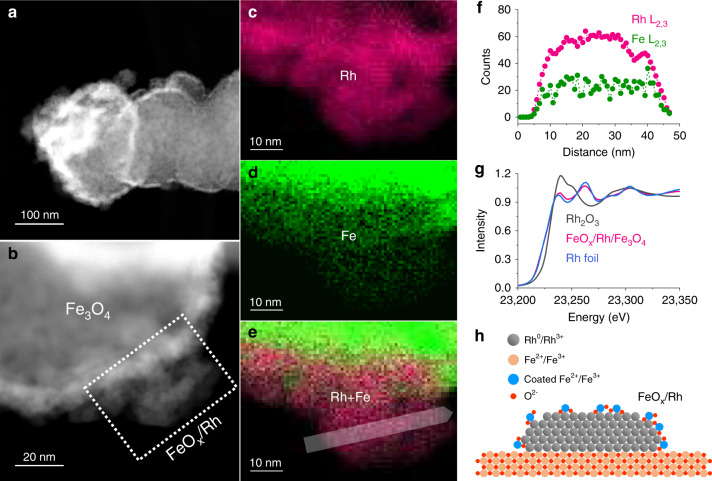
Structural characterization of FeO_*x*_/Rh/Fe_3_O_4_ showing the FeO_*x*_ coating on Rh. HAADF-STEM-EELS images of FeO_*x*_/Rh/Fe_3_O_4_ (**a**–**e**) and the corresponding line profile (**f**) showing mixed FeO_*x*_ and Rh domains. Rh K-edge XANES spectra (**g**) of FeO_*x*_/Rh/Fe_3_O_4_ suggesting Rh is mainly metallic and interacting with FeO_*x*_ species. **h** Scheme of the inverse structure on FeO_*x*_/Rh/Fe_3_O_4_.

We compared the inverse catalyst with Fe_3_O_4_-supported 1–2 nm Rh particles (Rh/Fe_3_O_4_) in CO_2_ hydrogenation. This reference was prepared by precipitating Rh^3+^ on Fe_3_O_4_ followed by treatment in air and reduction at 200 °C in H_2_ (Supplementary Fig. 8 for the Rh particle size distribution). To remove possible adsorbates remaining from synthesis and handling, the FeO_*x*_/Rh/Fe_3_O_4_-fresh material was treated at the same conditions as Rh/Fe_3_O_4_, yielding the material denoted as FeO_*x*_/Rh/Fe_3_O_4_. This material showed the same features of the parent FeO_*x*_/Rh/Fe_3_O_4_-fresh. That is, the dispersed FeO_*x*_ species still decorated the metallic Rh particles (Fig. 3a–f and Supplementary Fig. 9). The inverse FeO_*x*_/Rh nanostructure was unaltered by the heat treatment, in agreement with the lower surface energy of iron oxide (Fig. 3h and Supplementary Table 3), which tends to wet the Rh surface^13^.

**Fig. 4:**
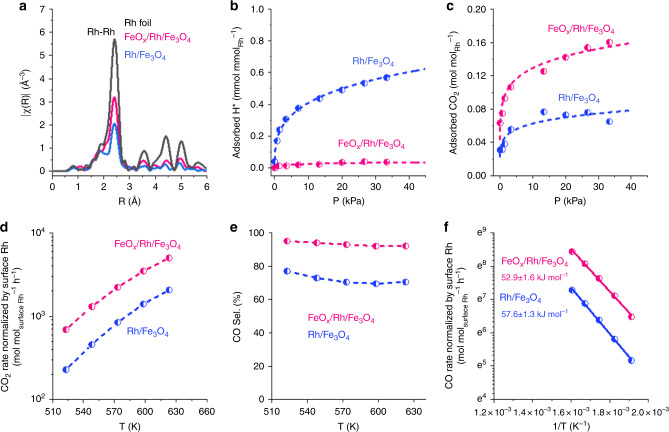
Characterizations and catalytic performance showing the impact of the FeO_*x*_ overlayer on CO_2_ reduction. Rh K-edge EXAFS spectra (**a**), isotherms of H_2_ and CO_2_ chemisorption (**b**, **c**), CO_2_ conversion rates (**d**), and CO selectivity for CO_2_ conversion (**e**), and the Arrhenius plots for CO_2_ reduction to CO (**f**) for FeO_*x*_/Rh/Fe_3_O_4_ and Rh/Fe_3_O_4_. Reaction conditions: 523–623 K, 101 kPa, CO_2_/H_2_/He = 7/28/105 mL min^−1^ (gas hourly space velocity of 7 × 10^5^ mL g^−1^ h^−1^). The CO_2_ and CO rates are normalized to the concentration of surface Rh as derived from EXAFS fitting.

According to XANES (Fig. 3g) and EXAFS results (Fig. 4a), Rh in both FeO_*x*_/Rh/Fe_3_O_4_ and Rh/Fe_3_O_4_ are mainly metallic with a Rh–Rh distance of 2.68 Å. The Rh–Rh coordination number for FeO_*x*_/Rh/Fe_3_O_4_ is ~8.9 while for Rh/Fe_3_O_4_ it is 6.7 (Supplementary Tables 4 and 5 and Supplementary Figs. 10 and 11). This suggests that the Rh dispersion of FeO_*x*_/Rh/Fe_3_O_4_ was lower than Rh/Fe_3_O_4_ (i.e., 56% and 82%, respectively) (Supplementary Fig. 12). The difference in Rh dispersion was supported by time-of-flight secondary ion mass spectrometry (TOF-SIMS), which showed more abundant Rh_2_O^+^ fragments for FeO_*x*_/Rh/Fe_3_O_4_ than for Rh/Fe_3_O_4_ (Supplementary Fig. 13).

In contrast to the EXAFS of FeO_*x*_/Rh/Fe_3_O_4_ showing 56% Rh dispersion, H_2_ chemisorption indicates that only 5.6% of Rh is available to adsorb H_2_ (Fig. 4b and Supplementary Table 6). The discrepancy between EXAFS and H_2_ chemisorption is clearly due to the presence of FeO_*x*_ overlayer. For Rh/Fe_3_O_4_, H_2_ chemisorption suggests a 70% dispersion, which is in good agreement with EXAFS results (i.e., most of the surface Rh atoms in the nanoparticles are available to adsorb H_2_). Both FeO_*x*_/Rh/Fe_3_O_4_ and Rh/Fe_3_O_4_ have the same adsorption equilibrium constant for H_2_ chemisorption (~7, Supplementary Table 6). Therefore, metallic Rh atoms are the sites for H_2_ activation on both materials.

### Catalytic improvement by the FeO_*x*_ overlayer

We targeted CO_2_ hydrogenation to test the activity of our inverse catalysts. Thus, we measured isotherms for CO_2_ adsorption (Fig. 4c), which showed that FeO_*x*_/Rh/Fe_3_O_4_ can adsorb more CO_2_ than Rh/Fe_3_O_4_ and pure Fe_3_O_4_ (Supplementary Table 6) (i.e., 7.2, 4.3, and 2.2 μmol_CO2_ g^−1^, respectively, at 33 kPa). The adsorption equilibrium constant for FeO_*x*_/Rh/Fe_3_O_4_ also is higher than for Rh/Fe_3_O_4_ (i.e., 100 and 51, respectively) (Fig. 4c and Supplementary Table 6). Thus, the adsorption sites on the inverse FeO_*x*_/Rh catalyst have stronger interactions with CO_2_ than the sites in Rh/Fe_3_O_4_. The differences in adsorption capacity and strength have important consequences in the coverages of molecular species during the reaction (Supplementary Table 7 and Supplementary Figs. 14 and 15), and thus the catalytic performance described below.

The inverse FeO_*x*_/Rh/Fe_3_O_4_ catalyst showed high activity for CO_2_ reduction per mol of surface Rh (determined from the Rh–Rh coordination number from EXAFS analysis) compared to that of Rh/Fe_3_O_4_ (Fig. 4d). We used this normalization to reflect the surface of the catalysts that is potentially active, i.e., Rh with or without interactions with the support (note however, these trends are the same per mass of catalyst and mass of Rh). The selectivity to CO and the corresponding CO production rates are also higher on FeO_*x*_/Rh/Fe_3_O_4_ than on Rh/Fe_3_O_4_ (Fig. 4e, f). This highlights the higher activity of the FeO_*x*_-coated particles than simple supported Rh particles. We analyzed the intrinsic activity of the materials not by normalizing rates to the fraction of exposed Rh (as determined from H_2_ chemisorption) nor to the fraction of Rh covered by oxide species (Supplementary Table 8 and Supplementary Note). Instead, we considered that the uptake of CO_2_ serves as titration of adsorption sites that can potentially produce CO (see the supporting information for more details). The rates of CO production normalized to the concentration of sites that chemisorb CO_2_ were, e.g., 1657 and 1222 h^−1^ on FeO_*x*_/Rh/Fe_3_O_4_ and Rh/Fe_3_O_4_, respectively, at 250 °C. The similarity of these values, and of the activation energies for CO production (Fig. 4f), allows us concluding that the highly active and selective sites in both systems are similar. These sites, in view of the negligible activity of SiO_2_-supported Rh and pure Fe_3_O_4_ (Supplementary Table 8) are undoubtedly identified as Rh–Fe_3_O_4_ interfaces^30–33^.

We also tested the FeO_*x*_/Rh nanoparticles (Fig. 1e) and the parent Rh nanoparticles in CO_2_ reduction (Supplementary Table 9). The FeO_*x*_/Rh nanoparticles were one order of magnitude more active than the bare Rh nanoparticles. The bare Rh nanoparticles produced both CO and methane in equimolar concentrations, while FeO_*x*_/Rh nanoparticles selectively yielded CO. These observations further support our claim that the FeO_*x*_ adlayers increase the activity for CO_2_ conversion and the selectivity to CO. The FeO_*x*_/Rh nanoparticles, however, led to 1–2 orders of magnitude lower rate for CO_2_ reduction than the inverse FeO_*x*_/Rh/Fe_3_O_4_ catalyst, which highlights the role of the Fe_3_O_4_ support, which maintains the FeO_*x*_/Rh nanoparticles separated.

The FeO_*x*_/Rh/Fe_3_O_4_ inverse catalyst is also more productive than typical supported noble-metal nanoparticles and atomically dispersed Rh (Supplementary Table 10). Thus, leaving some exposed Rh on the surface of FeO_*x*_/Rh/Fe_3_O_4_ does not lead to low activity because the surface behaves like Rh–Fe_3_O_4_ interfaces.

In summary, this galvanic replacement approach to prepare inverse FeO_*x*_/metal nanostructures not only yields particularly compelling catalytic reactivity under real conditions but is versatile and easily scalable^13,17,34^. The ability to control the surface functionality of metal nanoparticles enables a palette for catalyst design via galvanic replacement. The presence of the oxide overlayer makes the metal much more efficient for activating CO_2_ while maintaining its hydrogenation ability. That is, the whole surface of the metal particle functions as metal/oxide interface with redox sites for adsorbing CO_2_ near metal domains that dissociate H_2_ but with limited capacity to produce methane.

## Methods

### Materials

The chemicals including magnetite (Fe_3_O_4_) nanoparticles (50–100 nm), RhCl_3_ (37% Rh), rhodium (III) nitrate hydrate (Rh(NO_3_)_3_·H_2_O), FeCl_2_ (≥99.0%), FeCl_3_ (≥99.0%), urea (99.0–100.5%), polyvinylpyrrolidone (PVP), and ethylene glycol were purchased from Sigma-Aldrich. The deionized water was obtained from a Milli-Q water system.

### Synthesis of FeO_*x*_/Rh/Fe_3_O_4_-fresh, FeO_*x*_/Rh/Fe_3_O_4_ and FeO_*x*_/Pt/Fe_3_O_4_-fresh

The FeO_*x*_/Rh/Fe_3_O_4_-fresh with the pre-set Rh loading of 0.5 wt% was prepared by galvanic replacement between Rh^3+^ and partially reduced magnetite (Fe_3_O_3.7_). In a typical procedure, 9.95 g of Fe_3_O_4_ was reacted in 5 vol.% H_2_/N_2_ at 400 °C in a tube furnace to produce Fe_3_O_3.7_. The Fe_3_O_4_ symmetry group remained for Fe_3_O_3.7_ after this step (Supplementary Fig. 1). A 10 mL aqueous solution of RhCl_3_ at a concentration of 5 mg_Rh_ mL^−1^ was mixed with 90 mL deionized water at room temperature. The Fe_3_O_3.7_ was then added to the solution and stirred for 7 h. The resulting material was separated, washed with water, and dried at 80 °C overnight. The as-prepared material was calcined in air at 450 °C with a ramping rate of 2 °C/min. The inductively coupled plasma (ICP) analysis showed that the effective Rh loading in the final material was 0.37 wt%. Prior to the catalytic test, the sample was treated at 200 °C in H_2_. The purpose of heat treatments is to remove the possible surface ligands and surface-oxidized Rh species that remained during the synthesis. A material containing Pt (FeO_*x*_/Pt/Fe_3_O_4_-fresh) was prepared by the same method with aqueous solution of H_2_PtCl_6_ as the precursor and a pre-set Pt loading of 0.5 wt%.

The dynamic changes of the Fe^2+^ and Fe^3+^ concentrations in the aqueous fraction during the galvanic replacement synthesis (for FeO_*x*_/Rh/Fe_3_O_4_-fresh) were analyzed by the ferrozine method^28^. The suspension was centrifuged to isolate the aqueous fraction during the galvanic replacement (0, 0.1, 0.5, 1, 2, 3, 4, 5, 6, and 7 h). The pH values for the aqueous solutions increased slightly at first (from 4.0 to 5.0), and then decreased to ~2.5. The resulting aqueous solutions were diluted in a 10^−2^ M HCl solution and used for the analysis. The Fe^2+^ can react with ferrozine to form a stable magenta complex which gives a maximum absorbance at 562 nm on an ultraviolet–vis spectrophotometer. The Fe^3+^ fraction can be detected by reducing with hydroxylamine hydrochloride solution, stabilized in a buffer, and followed by complexing with ferrozine.

### Synthesis of Rh/Fe_3_O_4_

The Rh/Fe_3_O_4_ with a Rh loading of 0.5 wt% was prepared by a urea hydrolysis assisted deposition method. In a typical procedure, 9.95 g of Fe_3_O_4_ were dispersed in 100 mL deionized water. Then, a 10 mL aqueous solution of RhCl_3_ at a concentration of 5 mg_Rh_ mL^−1^ was added into the suspension and rigorously stirred for 12 h at room temperature. An excess of urea (urea/[Rh] molar ratio = 60) was added to the suspension for deposition of Rh^3+^. The Rh^3+^ can be deposited homogeneously and slowly with the help of urea hydrolysis in a hydrothermal condition (90 °C) for 6 h. The resulting material was separated, washed with water, and dried at 80 °C overnight. The as-prepared material was treated in air at 450 °C with a ramping rate of 2 °C min^−1^. The ICP results suggested that the Rh loading was 0.37 wt%. Prior to the catalytic test, the sample was treated at 200 °C in H_2_. A reference Rh/SiO_2_ with the Rh loading of 0.5 wt% was also prepared by the urea hydrolysis deposition method, followed by the same treatments before catalytic test.

### Synthesis of Rh nanoparticles (PVP method)

In a typical procedure, Rh nanoparticles were synthesized following a polyol-based method. Rh nitrate (Rh amount 100 mg) was dispersed in 60 mL of ethylene glycol in the presence of a stabilizer (PVP) and heated under reflux for 6 h. The Rh nanoparticles then were washed with acetone and water eight times before used for model synthesis experiments.

### Mixing of Rh^3+^ and Fe^2+^ cations in solution in the absence of solid

In a typical procedure, 0.25 mL RhCl_3_ aqueous solution (5 mg_[Rh]_ mL^−1^), 1 mL FeCl_2_ aqueous solution (2 mg_[Fe]_ mL^−1^), and 4 mL deionized water were mixed at room temperature and stirred for 7 h. This procedures were perfomed in a N_2_ glove box.

### Reaction of Rh^0^ nanoparticle and Fe^2+^ cations

After washing three times with deionized water, 1.25 mg Rh^0^ nanoparticles were dispersed in 4 mL deionized water and mixed with 1 mL FeCl_2_ aqueous solution (2 mg_[Fe]_ mL^−1^) at room temperature. The resulting suspension then was stirred for 7 h. The Rh^0^ nanoparticles immersed in Fe^2+^ solution and parent Rh^0^ nanoparticles were also diluted in SiO_2_ as the reference samples (Rh loading of 0.5%) for handling and catalytic testing.

### Reaction of Rh^0^ nanoparticles and Fe^3+^ cations

After washing three times with deionized, 1.25 mg Rh^0^ nanoparticles were dispersed in 4 mL deionized water and mixed with 1 mL FeCl_3_ aqueous solution (2 mg_[Fe]_ mL^−1^) at room temperature. The resulting suspension then was stirred for 7 h.

### Characterization

HAADF-STEM measurements were conducted with an aberration-corrected FEI Titan 80-300 STEM operated at 300 kV. EELS mapping and analysis were performed with aberration-corrected JEOL-ARM200F instrument operated at 200 kV. The instrument (Quantum 965) is capable of performing dual EELS experiment. The EELS mapping was performed in the STEM mode in the range of –50 to 500 eV for the zero-loss peak, 300 to 800 eV for the iron signal, and 2500 to 3500 eV for rhodium the signal maps. The zero-loss peak for zero-loss calibration was acquired in low loss spectrum images and aligned at 0 eV. The images and EELS data were analyzed and processed using Gatan Digital Micrograph software. The EELS maps were constructed by analyzing the Fe L_2,3_ (~708 eV), Rh L_2,3_ (~3004 eV), and Pt M_4,5_ (~2122 eV) edge peaks after the background subtraction.

X-ray absorption spectroscopy measurements were conducted in sector 20 of the Advanced Photon Source operated by Argonne National Laboratory. A rejection mirror was used to reduce the effects of harmonics. The metal foil was placed downstream of the sample cell, as a reference to calibrate the photon energy of each spectrum. The EXAFS spectra were analyzed with the ATHENA (*χ*(k) oscillation background removal), FEFF9 (theoretical model calculation), and ARTEMIS software packages. The fits to the Rh K-edge EXAFS *χ*(k) data were weighted by *k*^2^ and windowed between 1.5 Å^−1^ < k < 15.0 Å^−1^ using a Hanning window with dk = 1.0 Å^−1^.

H_2_ and CO_2_ chemisorption experiments were conducted with a Micromeritics 2020 instrument. In a typical procedure, 100 to 200 mg of the sample was degassed at 100 °C for 30 min, followed by in situ treatment at 200 °C in H_2_ and evacuation at 200 °C for 30 min. Then, the temperature was decreased to 35 °C under vacuum. Prior to the chemisorption experiments, the sample was further evacuated for 40 min. The adsorbates (H_2_ or CO_2_) were introduced into the system for the measurements of chemisorption isotherms. The first chemisorption isotherm was measured in the pressure range of 0–40 kPa at 35 °C. The sample was evacuated after the first adsorption cycle and a second chemisorption isotherm was recorded. The CO_2_ uptake on the parent Fe_3_O_4_ has been subtracted for plotting and derivation of adsorption parameters.

N_2_ physisorption experiments at −196 °C were performed on a Micromeritics 2020 instrument. The samples were degassed in vacuum at 200 °C before the measurements.

TOF-SIMS was applied with a TOF-SIMS V spectrometer (IONTOF GmbH, Münster, Germany) equipped with a 25 keV bismuth cluster ion source, a 20 keV Ar_*n*_^+^, and a 2 keV Cs^+^/O_2_^+^ sputtering ion sources. Prior to the TOF-SIMS experiments, the samples were deposited on an Au(111) substrate and exposed to ultrahigh vacuum overnight.

X-ray diffraction experiments were performed in a Philips X′pert Multi-Purpose Diffractometer equipped with a Cu anode (50 kV and 40 mA).

The elemental composition of samples was measured by ICP optical emission spectroscopy (Perkin Elmer 7300DV). Prior to the ICP experiments, the samples were digested in a mixture of HNO_3_/HCl/HF/H_2_O followed by H_3_BO_3_ addition for extra HF treatment.

### Reaction tests

The CO_2_ reduction was performed in a flow reactor equipped with an online gas chromatograph (Agilent 7890B). In a typical procedure, prior to the catalytic test, 12 mg of 30–80 mesh catalyst (diluted with 50 mg SiC) was loaded into the reactor and treated at 200 °C in 20 vol.% H_2_ with a ramping rate of 2 °C min^−1^. After the reactor reached the target reaction temperature, a mixture of CO_2_, H_2_, and He with a total flow rate of 140 mL min^−1^ was fed into the reactor (CO_2_:H_2_:He = 7:28:105).

### Correlations of coordination number and metal dispersion

The correlation between coordination number and metal dispersion was derived from the data in the reference (Supplementary Fig. 12)^35^. The relationship between the coordination number of metal–metal shell and the metal dispersion was derived based on two different shapes of metal particles (spherical and raft-like shapes).

### Calculation of adsorption constant and monolayer coverage from isotherms

The adsorption constant and monolayer coverage were derived from the chemisorption isotherms where chemisorption is treated as a chemical reaction between the gas-phase molecule (*A*) and the site (*) for adsorption (Eq. (5)).

5 $$A + \ast \, \rightleftarrows \, A \ast.$$

The adsorption can be fitted with a Langmuir adsorption model (Eq. (6)).

6 $$\theta _A = \frac{V}{{V_m}} = \frac{{\rm{{KP}}}}{{\rm{{1 + KP}}}}.$$

The adsorption parameters can be obtained from the linear form of Eq. (2) (Eq. (7)). In Eq. (7), *θ*_*A*_ is the fractional coverage of the adsorption sites, *P* is the partial pressure of the adsorbate, *V*_*m*_ is the volume of the monolayer, and *K* is the equilibrium adsorption constant.

7 $$\frac{1}{{\theta _A}} = V_m\left( {\frac{1}{V}} \right) = \frac{1}{K}\left( {\frac{1}{P}} \right) + 1.$$

## Supplementary information

Supplementary Information

## Data Availability

The source data underlying Figs. 1–4 are provided as a Source Data file. The other relevant data that support the findings of this study are available from the corresponding author upon request. Source Data are provided with this paper.

## Source data

Source Data
