# Supplementary material for: Inverse iron oxide/metal catalysts from galvanic replacement
Source: Nat Commun. 2020 Jun 29;11:3269. doi: 10.1038/s41467-020-16830-4 (PMC7324589; doi:10.1038/s41467-020-16830-4)
Supplement: Supplementary file 1 — Supplementary Information [file 41467_2020_16830_MOESM1_ESM.pdf]

Inverse Iron Oxide/Metal Catalysts from Galvanic Replacement

Y. Zhu et al.

Supplementary Material for

Inverse Iron Oxide/Metal Catalysts from Galvanic Replacement

Yifeng Zhu[1,2], Xin Zhang[2], Katherine Koh[1,2], Libor Kovarik[3], John L. Fulton[1,2],
Kevin M. Rosso[2], Oliver Y. Gutiérrez[1,2]*

Correspondence to: oliver.gutierrez@pnnl.gov

Supplementary Note 1. Analysis of the activity of $FeO_x/Rh/Fe_3O_4$.
Supplementary Figure 1. Comparison of X-ray diffraction spectra of Fe_3O_4 and $Fe_3O_{3.7}$, suggesting the magnetite symmetry group remained for $Fe_3O_{3.7}$.
Supplementary Figure 2. HAADF-STEM images of the $FeO_x/Rh/Fe_3O_4$ showing the uniformity of as formed nanostructures.
Supplementary Figure 3. Characterizations of $FeO_x/Rh/Fe_3O_4$-fresh.
Supplementary Figure 4. HAADF-STEM-EELS analysis of selected area of $FeO_x/Rh/Fe_3O_4$-fresh.
Supplementary Figure 5. HAADF-STEM-EELS analysis of selected area of $FeO_x/Rh/Fe_3O_4$-fresh.
Supplementary Figure 6. The EXAFS analysis of $FeO_x/Rh/Fe_3O_4$-fresh.
Supplementary Figure 7. HAADF-STEM-EELS analysis of selected area of $FeO_x/Pt/Fe_3O_4$-fresh.
Supplementary Figure 8. HAADF-STEM analysis of Rh/Fe_3O_4.
Supplementary Figure 9. Representative high-resolution HAADF-STEM images of Rh species on the $FeO_x/Rh/Fe_3O_4$.
Supplementary Figure 10. The EXAFS analysis of $FeO_x/Rh/Fe_3O_4$.
Supplementary Figure 11. The EXAFS analysis of Rh/Fe_3O_4.
Supplementary Figure 12. Correlation between the metal-metal coordination number and metal dispersion for metal nanoparticles summarized from reference (*1*).
Supplementary Figure 13. TOF-SIMS analysis of Rh/Fe_3O_4 and $FeO_x/Rh/Fe_3O_4$.
Supplementary Figure 14. Reaction orders in CO_2 for $FeO_x/Rh/Fe_3O_4$ and Rh/Fe_3O_4.
Supplementary Figure 15. Reaction orders in H_2 for $FeO_x/Rh/Fe_3O_4$ and Rh/Fe_3O_4.
Supplementary Table 1. Linear combination fitting of the XANES spectra of $FeO_x/Rh/Fe_3O_4$-fresh, $FeO_x/Rh/Fe_3O_4$ and Rh/Fe_3O_4.
Supplementary Table 2. EXAFS fitting parameters for $FeO_x/Rh/Fe_3O_4$-fresh.
Supplementary Table 3. Surface energies of Rh, Pt, and iron oxides reported in the literature.
Supplementary Table 4. EXAFS fitting parameters for $FeO_x/Rh/Fe_3O_4$.
Supplementary Table 5. EXAFS fitting parameters for Rh/Fe_3O_4.
Supplementary Table 6. Adsorption constants and monolayer coverages for H_2 and CO_2 on $FeO_x/Rh/Fe_3O_4$ and Rh/Fe_3O_4.
Supplementary Table 7. Activation energies for conversion of CO_2, production of CO, and production of CH_4 on $FeO_x/Rh/Fe_3O_4$ and Rh/Fe_3O_4.
Supplementary Table 8. Catalytic performance and normalized rates for CO production.
Supplementary Table 9. The comparison of the performance of $FeO_x/Rh/Fe_3O_4$, FeO_x/Rh NPs and Rh NPs at 250 ^oC.
Supplementary Table 10. Comparison of reaction rates of CO_2 reduction.

Supplementary Note 1. Analysis of the activity of $FeO_x/Rh/Fe_3O_4$.

We performed the reduction of CO_2 on the parent Fe_3O_4 and on a reference Rh/SiO_2 catalyst. The parent Fe_3O_4 and a reference Rh/SiO_2 give the CO_2 conversion rates 1-2 orders of magnitude lower than the Rh/Fe_3O_4 and $FeO_x/Rh/Fe_3O_4$. This is in line with the general agreement that efficient CO_2 reduction requires sites at interfaces between metals and reducible metal oxides and that the reducible oxide and metal (Fe_3O_4 and Rh in this case), separated are less active.(*5-8*) Thus, we can discard any significant contribution of pure Rh and bare Fe_3O_4 in the reaction over the complex materials.

To understand the structure of active centers, we compared a series of normalized rates for Rh/Fe_3O_4 and $FeO_x/Rh/Fe_3O_4$ based on the surface Rh, exposed Rh, surface FeO_x sites and sites for CO_2 adsorption (table s8). We resorted to a combination of EXAFS and H_2 chemisorption to estimate how much Rh is covered and/or interacting with FeO_x. We considered that the dispersion determined from EXAFS fitting corresponds to the fraction of Rh atoms at the surface of the particles. The dispersion determined from H_2 chemisorption corresponds to the fraction of exposed Rh available to chemisorb H_2. The differences between the dispersion estimated by EXAFS fitting and the dispersion estimated by H_2 chemisorption correspond to the fraction of metallic Rh that is covered by or interacting with FeO_x. In the case of $FeO_x/Rh/Fe_3O_4$ that fraction is attributed mostly to the overlayer, whereas in Rh/Fe_3O_4 the difference is attributed to interface interacting with Fe_3O_4.

Based on EXAFS (Rh at the surface of the particles), the rates are 656 h^{-1} and 175 h^{-1} over $FeO_x/Rh/Fe_3O_4$ and Rh/Fe_3O_4, respectively. This outcome supports the conclusion that the FeO_x-covered metal particles are more active than supported Rh. The rates calculated from the fraction of Rh “covered by FeO_x” are 750 h^{-1} and 1240 h^{-1} over $FeO_x/Rh/Fe_3O_4$ and Rh/Fe_3O_4,

respectively. The results indicate the active sites may involve both the Rh and FeO_x near the interface. The intrinsic activity of both, interfaces and FeO_x covered Rh particles may be strongly affected by the geometric arrangements of oxide and metals domains.

Therefore, we considered that the concentration of chemisorbed CO_2 (which we experimentally determined as shown in Fig. 4b) corresponds to the concentration of active sites for the reaction. The TOFs are 1806 h^{-1} and 1222 h^{-1} over FeO_x/Rh/Fe_3O_4 and Rh/Fe_3O_4, respectively. The similarity of the two values (as well as the similar activation energies for CO production) align with that the most active sites in both systems are similar. These sites, in view of the negligible activity of pure Rh and Fe_3O_4, are undoubtedly attributed to Rh-FeO_x interfacial domains.

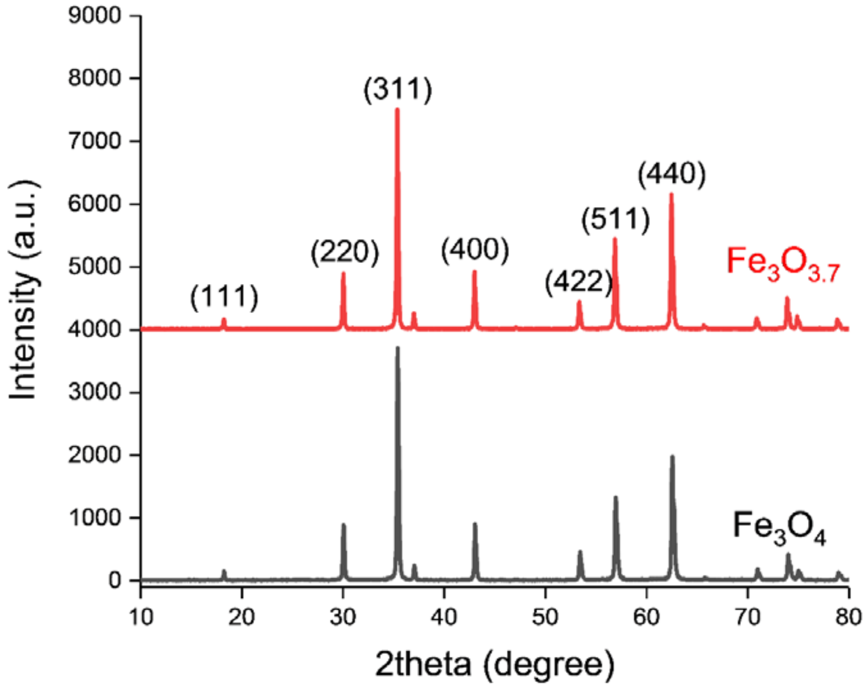

Supplementary Figure 1. Comparison of X-ray diffraction spectra of Fe_3O_4 and $Fe_3O_{3.7}$ suggesting the magnetite symmetry group remained for $Fe_3O_{3.7}$.

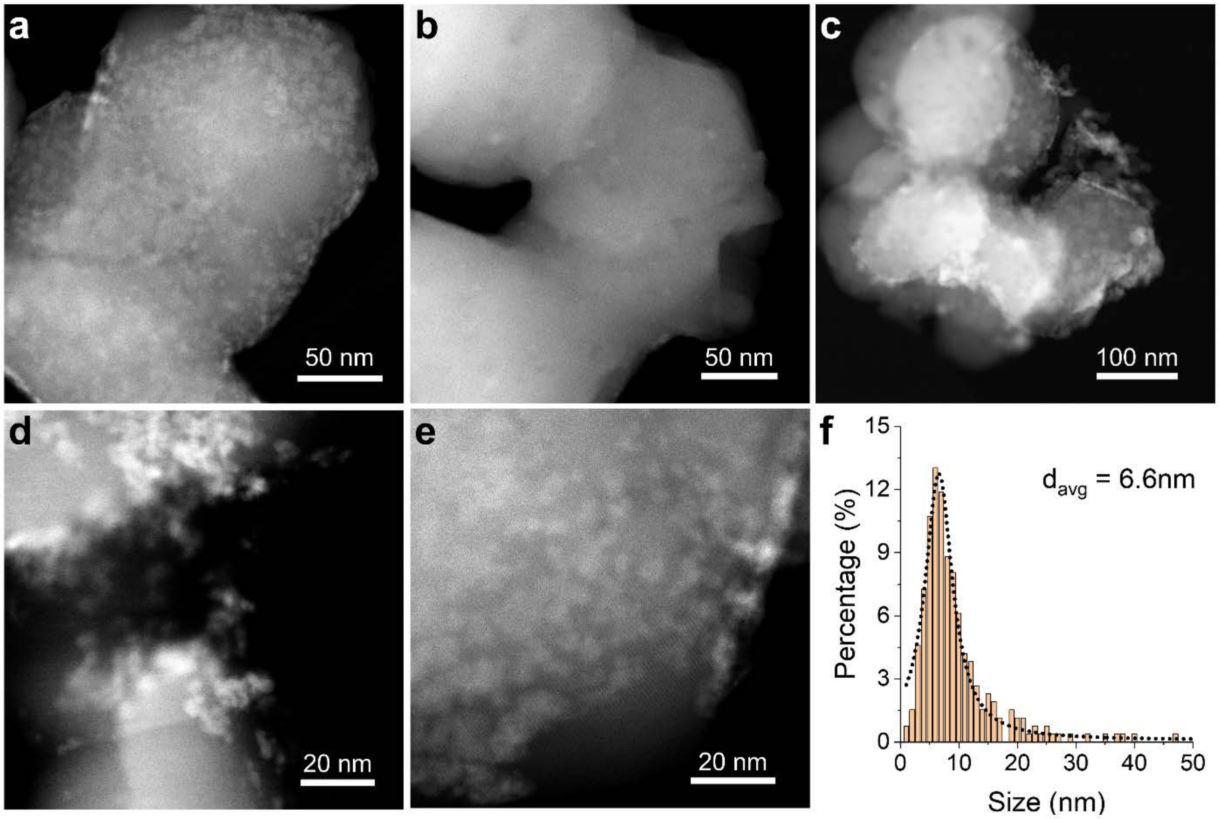

Supplementary Figure 2. HAADF-STEM images of $FeO_x/Rh/Fe_3O_4$ showing the uniformity of as-formed nanostructures. HAADF-STEM images (a-e) and the size distribution (f) of Rh cluster aggregates of the $FeO_x/Rh/Fe_3O_4$ catalyst.

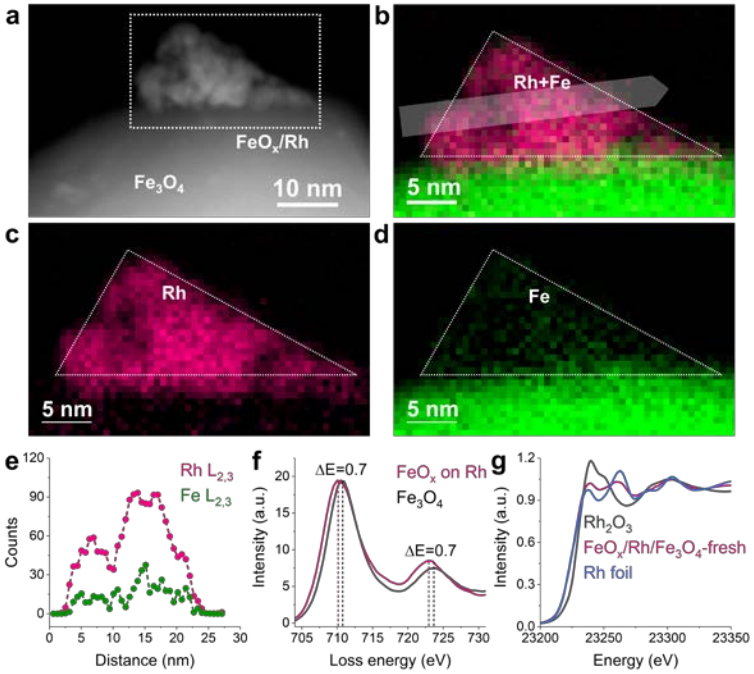

Supplementary Figure 3. Characterizations of $FeO_x/Rh/Fe_3O_4$-fresh. Representative HADDF-STEM image for $FeO_x/Rh/Fe_3O_4$-fresh (a); Rh and Fe L-edge EELS images of the selected area (b-d) and the corresponding EELS line-scan profile (e) showing the FeO_x coating on Rh. The Fe $L_{2,3}$ EELS spectra calibrated by zero-loss after background subtraction indicates that the Fe species on Rh has a valence state lower than +8/3 (f). Rh K-edge XANES (g).

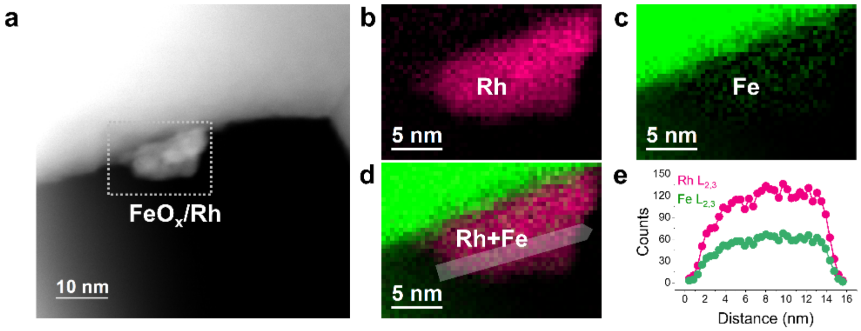

Supplementary Figure 4. HAADF-STEM-EELS analysis of a selected area of FeO_x/Rh/Fe_3O_4-fresh. Representative HADDF-STEM image for FeO_x/Rh/Fe_3O_4-fresh (a); Rh and Fe L-edge EELS images of the selected area (b-d) and the corresponding EELS line-scan profile (e) showing the FeO_x coating on Rh.

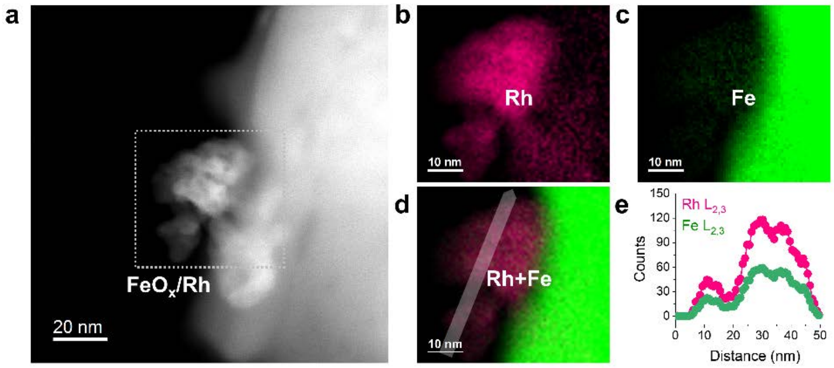

Supplementary Figure 5. HAADF-STEM-EELS analysis of a selected area of FeO_x/Rh/Fe_3O_4-fresh. Representative HADDF-STEM image for FeO_x/Rh/Fe_3O_4-fresh (a); Rh and Fe L-edge EELS images of the selected area (b-d) and the corresponding EELS line-scan profile (e) showing the FeO_x coating on Rh.

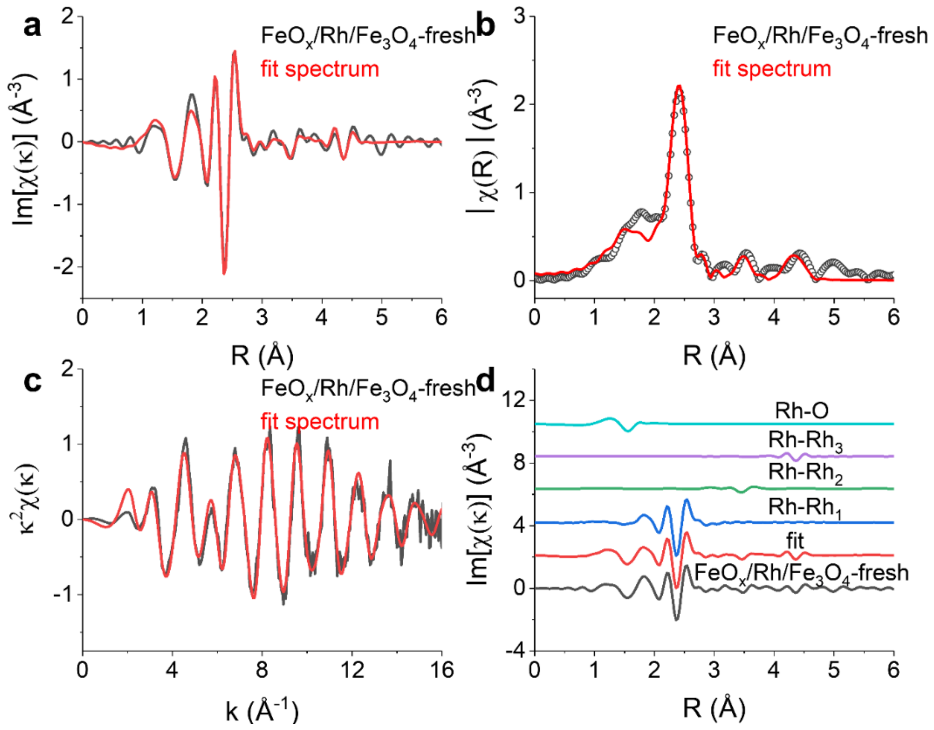

Supplementary Figure 6. EXAFS analysis of $FeO_x/Rh/Fe_3O_4$-fresh. The k^2-weighted Rh k-edge Fourier-transformed Im[χ(R)] (a), |χ(R)| (b) and χ(k) (c) spectra of the experimental and fit data for the $FeO_x/Rh/Fe_3O_4$-fresh. (d) The k^2-weighted Rh k-edge Fourier-transformed Im[χ(R)] of fitted paths for the $FeO_x/Rh/Fe_3O_4$-fresh.

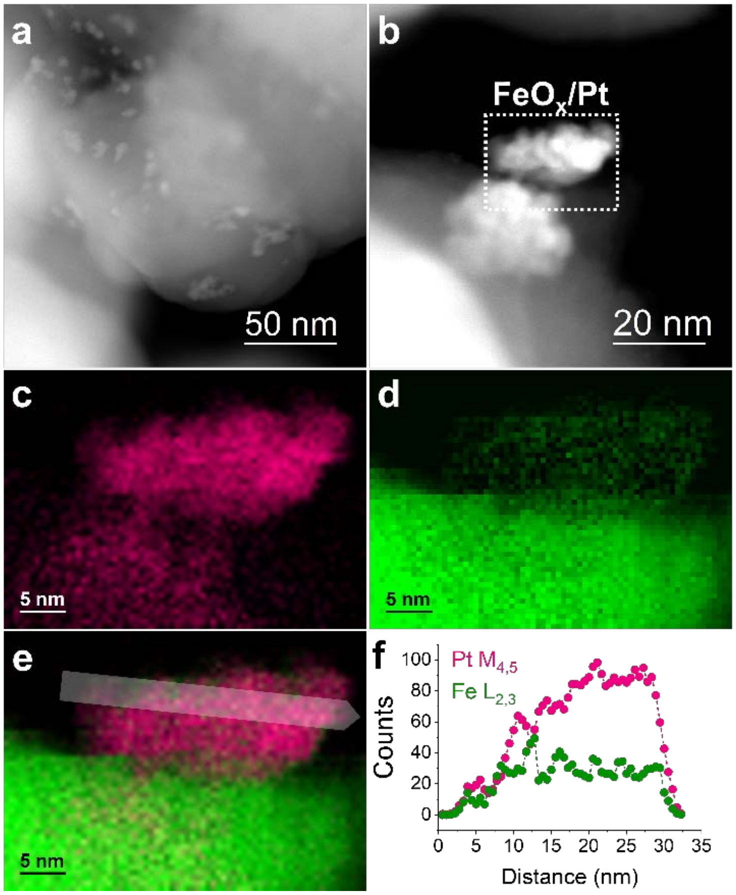

Supplementary Figure 7. HAADF-STEM-EELS analysis of a selected area of FeO_x/Pt/Fe_3O_4-fresh**.** HAADF-STEM images (a-b), the Pt M-edge, Fe L-edge and the mixed EELS images of the selected area (c-e) and the corresponding line profile (f) of FeO_x/Pt/Fe_3O_4-fresh showing that FeO_x is present on the Pt particles. The pink color represents the Pt M-edge and the green color represents the Fe L-edge.

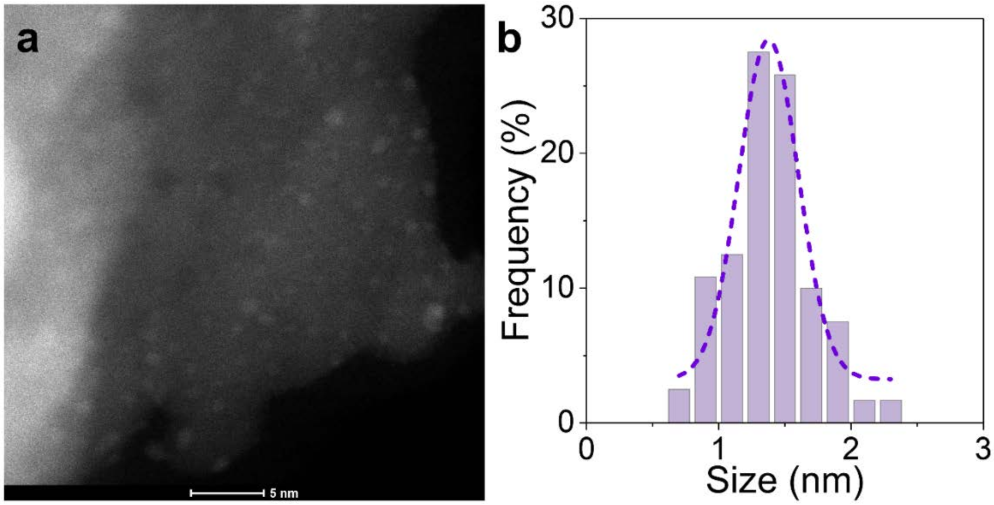

Supplementary Figure 8. HAADF-STEM analysis of Rh/Fe_3O_4. Representative HAADF-STEM image (a) and the particle size distribution (b) of Rh/Fe_3O_4.

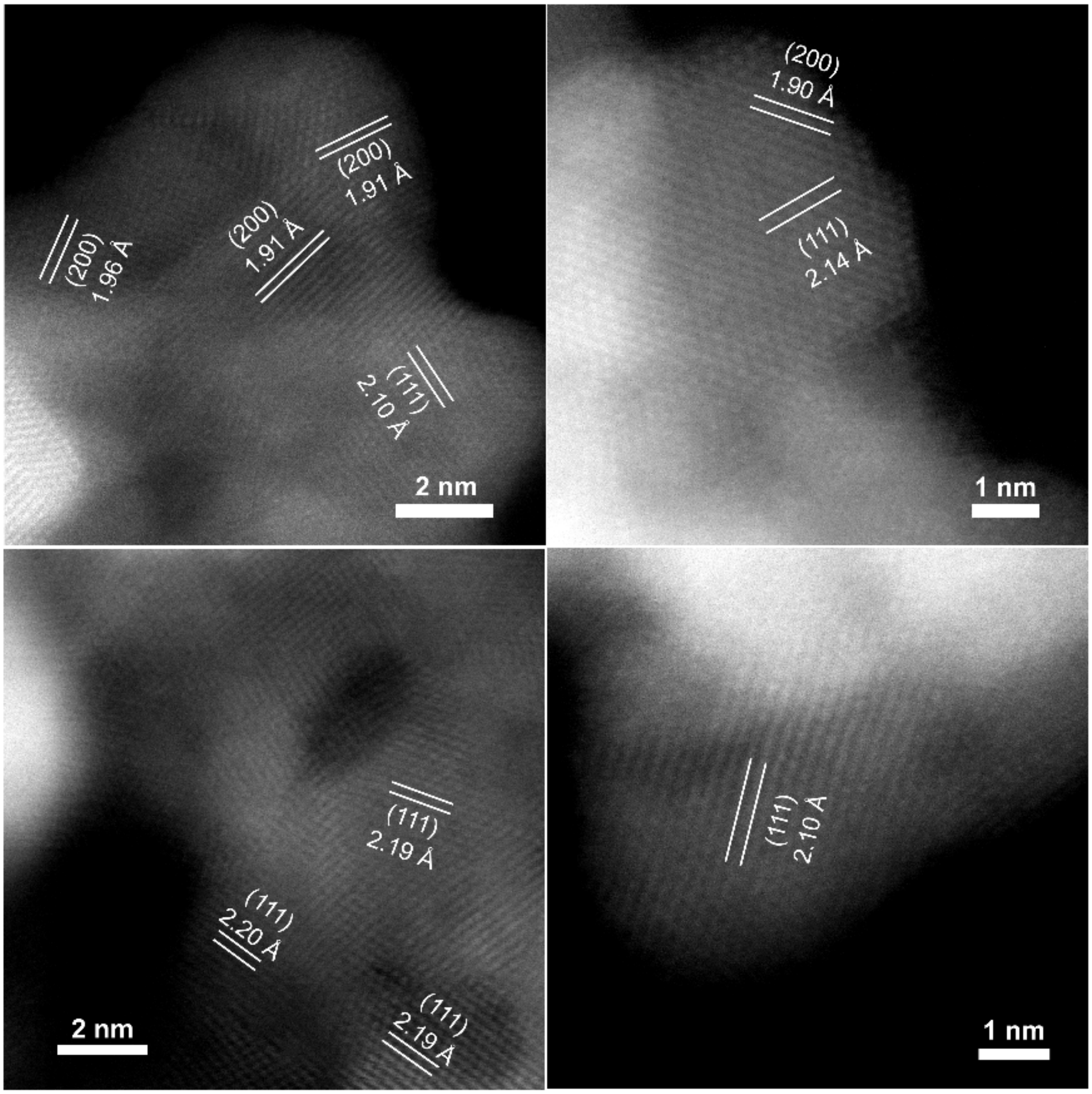

Supplementary Figure 9. Representative high-resolution HAADF-STEM images of Rh species on the $FeO_x/Rh/Fe_3O_4$.

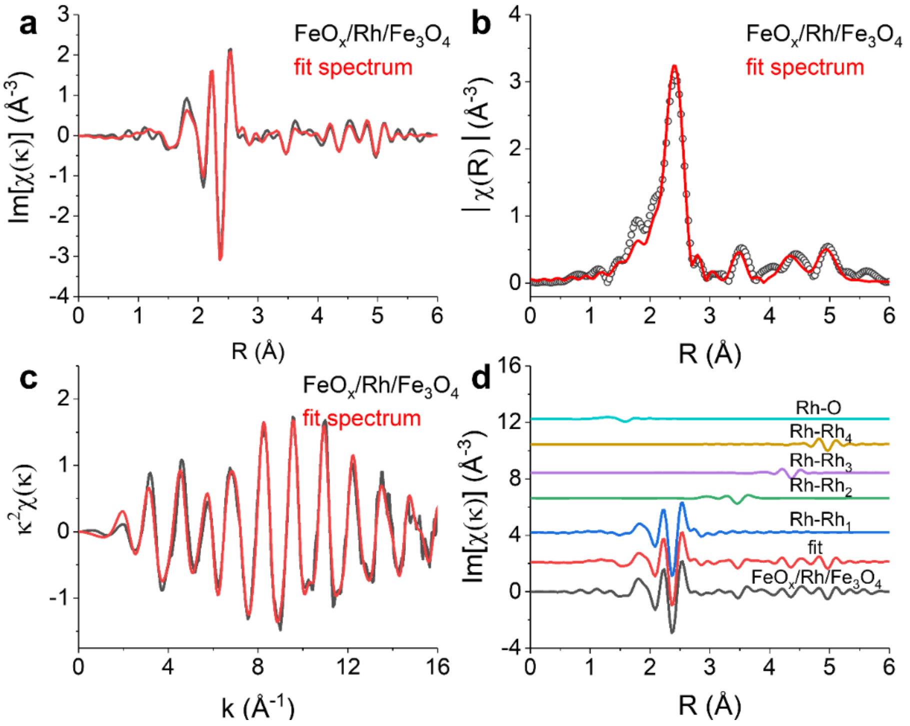

Supplementary Figure 10. EXAFS analysis of $FeO_x/Rh/Fe_3O_4$. The k^2-weighted Rh k-edge Fourier-transformed Im[χ(R)] (a), |χ(R)| (b) and χ(k) (c) spectra of the experimental and fit data for the $FeO_x/Rh/Fe_3O_4$. (d) The k^2-weighted Rh k-edge Fourier-transformed Im[χ(R)] of fitted paths for the $FeO_x/Rh/Fe_3O_4$.

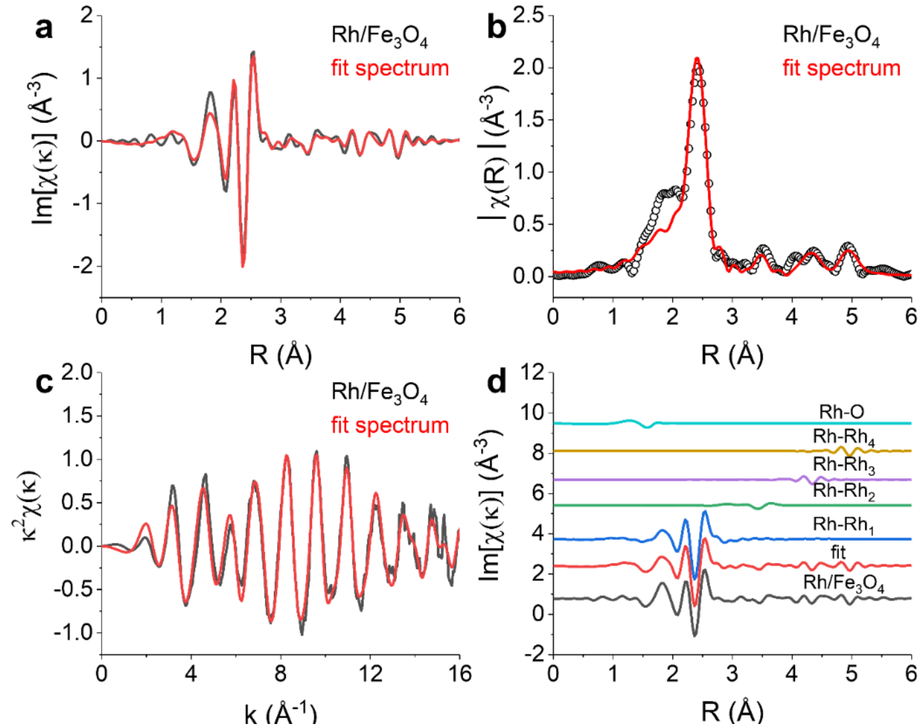

Supplementary Figure 11. EXAFS analysis of Rh/Fe_3O_4. The k^2-weighted Rh k-edge Fourier-transformed Im[χ(R)] (a), │χ(R)│ (b) and χ(k) (c) spectra of the experimental and fit data for the Rh/Fe_3O_4. (d) The k^2-weighted Rh k-edge Fourier-transformed Im[χ(R)] of fitted paths for the Rh/Fe_3O_4.

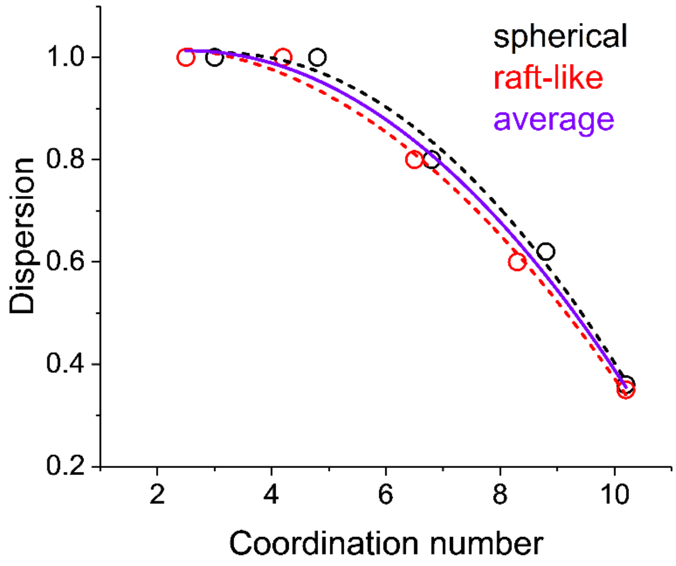

Supplementary Figure 12. Correlation between the metal-metal coordination number and metal dispersion for metal nanoparticles summarized from reference (*1*). Black is the spherical nanoparticle, red is the raft-like metal nanoparticles, and purple is the averaged correlation of spherical and raft-like nanoparticles.

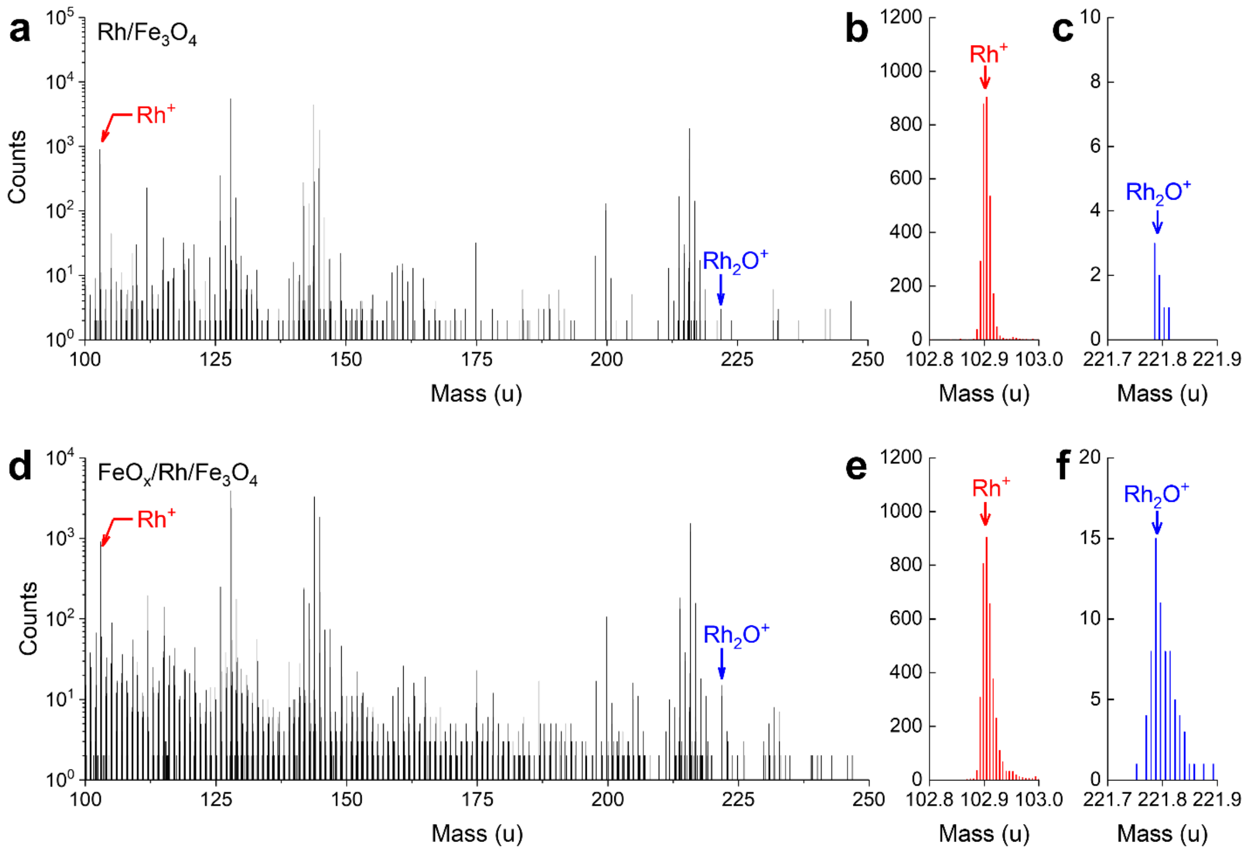

Supplementary Figure 13. TOF-SIMS analysis of Rh/Fe_3O_4 and $FeO_x/Rh/Fe_3O_4$. TOF-SIMS patterns for Rh/Fe_3O_4 (a-c) and $FeO_x/Rh/Fe_3O_4$ (d-f) suggesting that the Rh particle size in $FeO_x/Rh/Fe_3O_4$ is larger than that in Rh/Fe_3O_4.

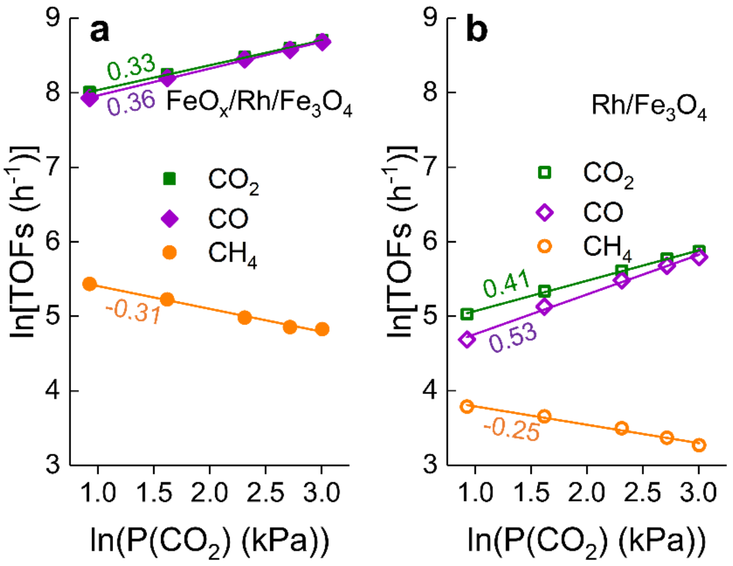

Supplementary Figure 14. Reaction orders in CO_2 for $FeO_x/Rh/Fe_3O_4$ and Rh/Fe_3O_4. Reaction orders in CO_2 measured for CO_2 conversion, CO and CH_4 formation at 250°C on $FeO_x/Rh/Fe_3O_4$ (a) and Rh/Fe_3O_4 (b). The orders in CO_2 for CO production on $FeO_x/Rh/Fe_3O_4$ were less positive than on Rh/Fe_3O_4 (e.g., 0.36 and 0.53 respectively), suggesting higher coverage of activated CO_2 on the inverse FeO_x/Rh than on supported Rh nanoparticles with only the perimeters contacting Fe_3O_4. These coverage differences agree well with the performance of the materials toward CO_2 chemisorption (i.e., the FeO_x-Rh sites have stronger affinity to CO_2 than metallic Rh sites).

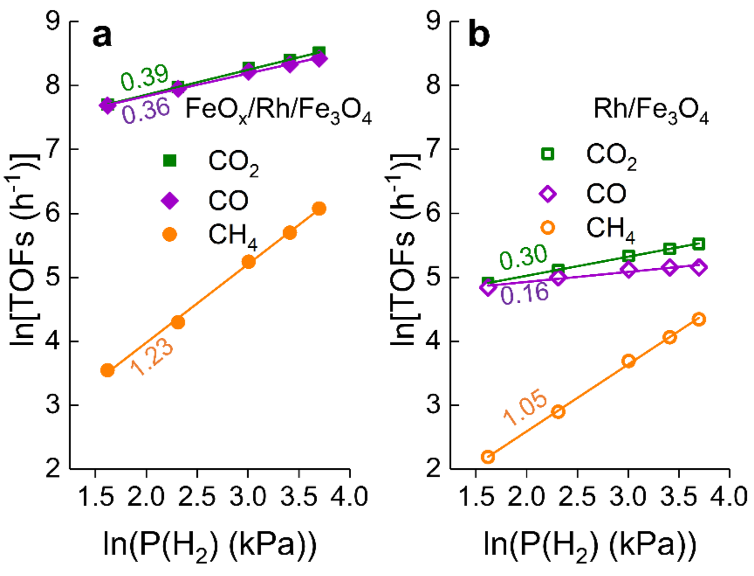

Supplementary Figure 15. Reaction orders in H_2 for $FeO_x/Rh/Fe_3O_4$ and Rh/Fe_3O_4. Reaction orders in H_2 measured for CO_2 conversion, CO and CH_4 formation at 250°C on $FeO_x/Rh/Fe_3O_4$ (a) and Rh/Fe_3O_4 (b). The reaction orders in H_2 were higher on $FeO_x/Rh/Fe_3O_4$ than on Rh/Fe_3O_4 (i.e., 0.36 and 0.16 for CO production, respectively). This reveals that the coverage of adsorbed H_2 is lower on the FeO_x-Rh interfaces than on the bare Rh particles. These coverage differences agree well with the performance of the materials toward H_2 chemisorption (i.e., the FeO_x-Rh sites have lower to H_2 than metallic Rh sites).

Samples	Rh^0(%)	$Rh^{\sigma+}$(%)
$FeO_x/Rh/Fe_3O_4$-fresh	77.3	22.7
$FeO_x/Rh/Fe_3O_4$	90.4	9.6
Rh/Fe_3O_4	90.2	9.8

Supplementary Table 1. Linear combination fitting of the XANES spectra of $FeO_x/Rh/Fe_3O_4$-fresh, $FeO_x/Rh/Fe_3O_4$ and Rh/Fe_3O_4.

Backscatterer	Length (Å)	Coordination number	Debye-Waller factor ($\Delta\sigma^2$, Å^2)
Rh-O	2.02 (±0.02)	1.9 (±0.5)	0.0056
$Rh-Rh_1$	2.68 (±0.01)	7.2 (±0.6)	0.0058
$Rh-Rh_2$	3.76 (±0.05)	5.9 (±3.4)	0.0137
$Rh-Rh_3$	4.66 (±0.001)	4.5 (±3.1)	0.0057

Supplementary Table 2. EXAFS fitting parameters for $FeO_x/Rh/Fe_3O_4$-fresh.

Materials	Surface energies (J/m^2)
Rh	2.66-2.70 (*2*)
Pt	2.48-2.49 (*2*)
Fe	1.91-2.32 (*3*)
$Fe_{1-x}O$ (wüstite)	0.59-1.05 (*4*)
Fe_3O_4 (magnetite)	0.36-0.4 (*4*)

Supplementary Table 3. Surface energies of Rh, Pt, and iron oxides reported in the literature.

Backscatterer	Length (Å)	Coordination number	Debye-Waller factor ($\Delta\sigma^2$, Å^2)
Rh-O	2.03 (±0.01)	0.6 (±0.3)	0.001824 (set)
$Rh-Rh_1$	2.68 (±0.01)	8.9 (±0.7)	0.0048
$Rh-Rh_2$	3.76 (±0.04)	6.4 (±3.7)	0.0102
$Rh-Rh_3$	4.66 (±0.02)	6.4 (±4.5)	0.0057
$Rh-Rh_4$	5.26 (±0.12)	6.9 (±4.6)	0.0032

Supplementary Table 4. EXAFS fitting parameters for $FeO_x/Rh/Fe_3O_4$.

Backscatterer	Length (Å)	Coordination number	Debye-Waller factor ($\Delta\sigma^2$, Å^2)
Rh-O	2.02 (±0.01)	0.7 (±0.5)	0.0033
Rh-Rh_1	2.67 (±0.01)	6.8 (±0.7)	0.0058
Rh-Rh_2	3.74 (±0.07)	5.1 (±4.0)	0.0152
Rh-Rh_3	4.64 (±0.02)	2.1 (±2.3)	0.0032
Rh-Rh_4	5.25 (±0.12)	2.7 (±2.9)	0.0023

Supplementary Table 5. EXAFS fitting parameters for Rh/Fe_3O_4.

Materials	$K[CO_2]$	$V_m[CO_2]$ (mol/mol$_{total\ Rh}$)	$K[H_2]$	$V_m[H]$ (mol/mol$_{total\ Rh}$)
$FeO_x/Rh/Fe_3O_4$	100.1	0.202	6.6	0.056
Rh/Fe_3O_4	51.4	0.068	7.3	0.702

Supplementary Table 6. Adsorption constants and monolayer coverages for H_2 and CO_2 on $FeO_x/Rh/Fe_3O_4$ and Rh/Fe_3O_4. The isotherms of $FeO_x/Rh/Fe_3O_4$ and Rh/Fe_3O_4 was subtracted by that of Fe_3O_4 before derivation of adsorption parameters.

Materials	$E_a[CO_2]$ (kJ/mol)	$E_a[CO]$ (kJ/mol)	$E_a[CH_4]$ (kJ/mol)
$FeO_x/Rh/Fe_3O_4$	53.8 (±1.7)	52.9 (±1.6)	67.3 (±3.7)
Rh/Fe_3O_4	60.1 (±2.0)	57.6 (±1.3)	67.0 (±4.2)

Supplementary Table 7. Activation energies for conversion of CO_2, production of CO, and production of CH_4 on $FeO_x/Rh/Fe_3O_4$ and Rh/Fe_3O_4. The activation energies for CO and methane production (53 to 58 kJ mol^{-1} and 67 kJ mol^{-1}, respectively) were similar on both materials. This points to invariant reaction routes over both catalysts, albeit with a small contribution from CH_4 production over $FeO_x/Rh/Fe_3O_4$.

Samples	CO_2 conversion rate mmol g^{-1}_{cat} h^{-1}	CO sel. (%)	CO rates based on surface Rh from EXAFS fitting	CO rates based on exposed Rh from H_2 chemisorption	CO rates based on the difference of EXAFS and H_2 chemisorption	CO rates based on amount of CO_2 chemisorbed at 33 kPa
$FeO_x/Rh/Fe_3O_4$	13.8	95.0	656	6558	750	1806
Rh/Fe_3O_4	6.8	77.0	175	204	1240	1222
Fe_3O_4	0.3	100.0	-	-	-	148
Rh/SiO_2	0.4	38.9	n.d.	5	n.d.	68

Supplementary Table 8. Catalytic performance and normalized rates for CO production. Rh/SiO_2 reference material was prepared by urea-hydrolysis deposition of Rh on silica in a solution of $RhCl_3$ followed by treatment in air and reduction at 200°C in H_2. The Rh content was 0.43 wt.% and the CO_2 uptake was $2.3x10^{-3}$ mol_{CO2}/g.

Samples	Rh loading (wt. %)	CO_2 rate (mmol/g_{cat}/h)	CO sel. (%)
$FeO_x/Rh/Fe_3O_4$	0.37	13.8	95.0
FeO_x/Rh nanoparticles	0.38	1.1	97.0
Rh nanoparticles	0.42	0.1	51.0

Supplementary Table 9. The comparison of the performance of $FeO_x/Rh/Fe_3O_4$, FeO_x/Rh NPs and Rh NPs at 250 ^oC. The nanoparticles were diluted in SiO_2 for handling. The preset Rh contents is 0.5 wt. %, same as $FeO_x/Rh/Fe_3O_4$.

No	Catalysts	Metal loading (wt. %)	Surface area (m^2/g)	Proportion of exposed metal (%)	Conditions	TOF (h^{-1})	E_a (kJ/mol)	Ref.
1	Ru/SiO_2	2.0	490	96.0	623 K $CO_2/H_2=0.1$	205	n.d.	(*9*)
2	Pt/Al_2O_3	0.97	146	63.7	573 K $CO_2/H_2=0.7$	37	n.d.	(*10*)
3	Pt/TiO_2(*10*)	0.98	132	18.2	573 K $CO_2/H_2=0.7$	359	n.d.	(*10*)
4	Ru/Al_2O_3	0.1	n.d.	100	623 K $CO_2/H_2=0.33$	403	82(CO) 62(CH_4)	(*11*)
5	Pd/Al_2O_3	0.5	200[a]	100	553 K $CO_2/H_2=0.25$	33	44-52	(*12*)
6	Pd/Al_2O_3	5	200[a]	11.0	553 K $CO_2/H_2=0.25$	107	37-52	(*12*)
7	$Pt_1Mo_{0.45}/SiO_2$	4.3	n.d.	3.9	473 K $CO_2/H_2=0.5$	1374	60.3±1.3	(*13*)
8	Pt/L	0.27	329	80.9	598 K $CO_2/H_2=1$	504	60±1.2	(*14*)
9	K_{80}-Pt/L	0.26	72	66.6	598 K $CO_2/H_2=1$	3024	50±1.5	(*14*)
10	Pt-K/mullite	2	46[a]	27.0	613 K $CO_2/H_2=1$	2016	34±1.3	(*15*)
11	Ru/SiO_2	0.4	968	n.d.	623 K $CO_2/H_2=0.25$	144	81±3	(*16*)
12	Au/UiO-67	2.4	725	n.d.	681 K $CO_2/H_2=0.33$	22	n.d.	(*17*)
13	Rh/Fe_3O_4 (this work)	0.37	23	70.2[b]	523 K $CO_2/H_2=0.25$	266	60.1 (±2.0)	–
14	Rh/Fe_3O_4 (this work)	0.37	23	70.2[b]	623 K $CO_2/H_2=0.25$	2407	60.1 (±2.0)	–
15	$FeO_x/Rh/Fe_3O_4$ (this work)	0.37	15	5.6[b]	523 K $CO_2/H_2=0.25$	6903	53.8 (±1.7)	–
16	$FeO_x/Rh/Fe_3O_4$ (this work)	0.37	15	5.6[b]	623 K $CO_2/H_2=0.25$	50025	53.8 (±1.7)	–

[a] the surface area of the parent supports. [b] measured by H_2 chemisorption.

Supplementary Table 10. Comparison of reaction rates of CO_2 reduction.

Supplementary References

1. B. J. Kip, F. B. M. Duivenvoorden, D. C. Koningsberger, R. Prins, Determination of metal particle size of highly dispersed Rh, Ir, and Pt catalysts by hydrogen chemisorption and EXAFS. *J. Catal.* **105**, 26-38 (1987).
2. L. Vitos, A. V. Ruban, H. L. Skriver, J. Kollár, The surface energy of metals. *Surf. Sci.* **411**, 186-202 (1998).
3. H. Jones, The Surface Energy of Solid Metals. *Metal Sci. J.* **5**, 15-18 (1971).
4. S. H. Overbury, P. A. Bertrand, G. A. Somorjai, Surface composition of binary systems. Prediction of surface phase diagrams of solid solutions. *Chem. Rev.* **75**, 547-560 (1975).
5. C.-Y. Chou, J. A. Loiland, R. F. Lobo, Reverse Water-Gas Shift Iron Catalyst Derived from Magnetite. *Catalysts* **9**, 773 (2019).
6. M. Amoyal, R. Vidruk-Nehemya, M. V. Landau, M. Herskowitz, Effect of potassium on the active phases of Fe catalysts for carbon dioxide conversion to liquid fuels through hydrogenation. *J. Catal.* **348**, 29-39 (2017).
7. N. Utsis, M. V. Landau, A. Erenburg, R. V. Nehemya, M. Herskowitz, Performance of Reverse Water Gas Shift on Coprecipitated and C-Templated BaFe-Hexaaluminate: The Effect of Fe Loading, Texture, and Promotion with K. *ChemCatChem* **10**, 3795-3805 (2018).
8. A. A. Hakeem *et al.*, The role of rhodium in the mechanism of the water–gas shift over zirconia supported iron oxide. *J. Catal.* **313**, 34-45 (2014).
9. S. Scirè, C. Crisafulli, R. Maggiore, S. Minicò, S. Galvagno, Influence of the support on CO_2 methanation over Ru catalysts: an FT-IR study. *Catal. Lett.* **51**, 41-45 (1998).
10. S. S. Kim, H. H. Lee, S. C. Hong, A study on the effect of support's reducibility on the reverse water-gas shift reaction over Pt catalysts. *Appl. Catal. A: Gen.* **423-424**, 100-107 (2012).
11. J. H. Kwak, L. Kovarik, J. Szanyi, CO_2 Reduction on Supported Ru/Al_2O_3 Catalysts: Cluster Size Dependence of Product Selectivity. *ACS Catal.* **3**, 2449-2455 (2013).
12. X. Wang, H. Shi, J. H. Kwak, J. Szanyi, Mechanism of CO_2 Hydrogenation on Pd/Al_2O_3 Catalysts: Kinetics and Transient DRIFTS-MS Studies. *ACS Catal.* **5**, 6337-6349 (2015).
13. I. Ro *et al.*, Measurement of intrinsic catalytic activity of Pt monometallic and Pt-MoO_x interfacial sites over visible light enhanced $PtMoO_x$ /SiO_2 catalyst in reverse water gas shift reaction. *J. Catal.* **344**, 784-794 (2016).
14. X. Yang *et al.*, Promotion effects of potassium on the activity and selectivity of Pt/zeolite catalysts for reverse water gas shift reaction. *Appl. Catal. B: Environ.* **216**, 95-105 (2017).
15. B. Liang *et al.*, Promoting role of potassium in the reverse water gas shift reaction on Pt/mullite catalyst. *Catal. Today* **281**, 319-326 (2017).
16. J. Dou, Y. Sheng, C. Choong, L. Chen, H. C. Zeng, Silica nanowires encapsulated Ru nanoparticles as stable nanocatalysts for selective hydrogenation of CO_2 to CO. *Appl. Catal. B: Environ.* **219**, 580-591 (2017).
17. H. Xu, Y. Li, X. Luo, Z. Xu, J. Ge, Monodispersed gold nanoparticles supported on a zirconium-based porous metal–organic framework and their high catalytic ability for the reverse water–gas shift reaction. *Chem. Commun.* **53**, 7953-7956 (2017).
